# Supplementary material for: Inequalities in colorectal cancer screening uptake in Wales: an examination of the impact of the temporary suspension of the screening programme during the COVID-19 pandemic
Source: BMC Public Health. 2023 Mar 22;23:546. doi: 10.1186/s12889-023-15345-z (PMC10031708; doi:10.1186/s12889-023-15345-z)
Supplement: Supplementary file 1 — Supplementary material (Tables 1S-3S) [file 12889_2023_15345_MOESM1_ESM.docx]

# Supplementary material (Tables 1S-3S)

| **Table 1S.** Univariable and multivariable analysis of BSW uptake for invitations during period 2019/20 (Invitation period 1^st^ August-31^st^ October) | | | | | | | | | |
| --- | --- | --- | --- | --- | --- | --- | --- | --- | --- |
| Characteristic | Population (n) | Uptake (%) | OR | 95% CI | p | Adjusted uptake (%) | aOR | 95% CI | p |
| **Overall** | 69,397 | 62.7 | - | - | - | 63.0 | - | - | **-** |
| **Sex**  Male  Female | 33,771  35,626 | 61.8  63.6 | Reference  0.92 | 0.90-0.95 | **<0.001** | 62.4  63.3 | Reference  1.04 | 1.01-1.07 | **0.022** |
| **Age group**  60-64 years  65-69 years  70-74 years | 28,482  18,532  22,383 | 58.5  62.8  68.0 | Reference  1.96  1.50 | 1.15-1.24  1.45-1.56 | **<0.001**  **<0.001** | 59.2  62.9  67.4 | Reference  1.17  1.44 | 1.13-1.22  1.38-1.49 | **<0.001**  **<0.001** |
| **Location**  Urban  Rural | 23,610  42,220 | 61.8  64.8 | Reference  1.14 | 1.10-1.18 | **<0.001** | 62.4  63.7 | Reference  1.09 | 1.05-1.12 | **<0.001** |
| **Income deprivation**  Q5 (least deprived)  Q4  Q3  Q2  Q1 (most deprived) | 14,380  14,427  13,723  12,529  10,771 | 69.4  66.0  63.0  59.4  53.9 | Reference  0.86  0.75  0.65  0.52 | 0.82-0.90  0.72-0.79  0.61-0.68  0.49-0.54 | **<0.001**  **<0.001**  **<0.001**  **<0.001** | 69.0  65.7  62.9  59.6  54.6 | Reference  0.86  0.76  0.66  0.54 | 0.81-0.90  0.72-0.80  0.62-0.69  0.51-0.56 | **<0.001**  **<0.001**  **<0.001**  **<0.001** |
| **Ethnic group**  White  Mixed  Asian  Black  Other  Unknown | 57,281  210  520  117  101  11,168 | 65.1  54.8  55.8  48.7  55.4  51.2 | Reference  0.65  0.68  0.51  0.67  0.56 | 0.49-0.85  0.56-0.81  0.35-0.73  0.45-0.99  0.54-0.59 | **0.002**  **<0.001**  **<0.001**  **0.044**  **<0.001** | 65.0  55.1  56.7  52.4  55.8  51.7 | Reference  0.66  0.71  0.60  0.68  0.57 | 0.50-0.88  0.60-0.85  0.41-0.88  0.45-1.02  0.55-60.0 | **0.004**  **<0.001**  **0.009**  0.062  **<0.001** |

| **Table 2S**. Univariable and multivariable analysis of BSW uptake for invitations during period 2018/19 (Invitation period 1^st^ August-31^st^ October) | | | | | | | | | |
| --- | --- | --- | --- | --- | --- | --- | --- | --- | --- |
| Characteristic | Population (n) | Uptake (%) | OR | 95% CI | p | Adjusted uptake (%) | aOR | 95% CI | p |
| **Overall** | 70,369 | 52.8 | - | - | **-** | 53.2 | - | - | **-** |
| **Sex**  Male  Female | 34,576  35,793 | 51.3  54.3 | Reference  0.89 | 0.86-0.91 | **<0.001** | 51.7  54.1 | Reference  1.10 | 1.07-1.13 | **<0.001** |
| **Age group**  60-64 years  65-69 years  70-74 years | 27,960  23,584  18,825 | 51.3  53.4  54.3 | Reference  1.09  1.13 | 1.05-1.13  1.08-1.17 | **<0.001** | 51.9  53.4  53.8 | Reference  1.07  1.08  1.10 | 1.03-1.10  1.04-1.12  1.07-1.13 | **<0.001**  **<0.001**  **<0.001** |
| **Location**  Urban  Rural | 23,881  43,254 | 52.2  54.3 | Reference  1.09 | 1.06-1.13 | **<0.001** | 52.8  53.1 | Reference  1.04 | 1.01-1.08 | **0.012** |
| **Income deprivation**  Q5 (least deprived)  Q4  Q3  Q2  Q1 (most deprived) | 14,190  14,843  14,086  12,804  11,212 | 60.1  55.5  52.9  49.5  44.4 | Reference  0.83  0.75  0.65  0.53 | 0.79-0.87  0.71-0.78  0.62-0.68  0.50-0.56 | **<0.001**  **<0.001**  **<0.001**  **<0.001** | 55.5  52.8  49.6  44.7 | Reference  0.84  0.75  0.66  0.53 | 0.80-0.88  0.71-0.79  0.63-0.69  0.51-0.56 | **<0.001**  **<0.001**  **<0.001**  **<0.001** |
| **Ethnic group**  White  Mixed  Asian  Black  Other  Unknown | 58,664  198  538  103  94  10,772 | 54.9  48.5  45.2  44.7  47.9  42.1 | Reference  0.77  0.58  0.66  0.76  0.59 | 0.59-1.02  0.57-80.3  0.45-0.98  0.50-1.13  0.57-0.62 | 0.072  **<0.001**  **0.039**  0.174  **<0.001** | 54.9  50.3  44.9  48.4  50.0  42.3 | Reference  0.83  0.68  0.78  0.82  0.60 | 0.62-1.11  0.57-0.81  0.52-1.17  0.54-1.24  0.58-0.63 | 0.207  **<0.001**  0.227  0.335  **<0.001** |

| **Table 3S.** Univariable and multivariable analysis of BSW uptake for invitations during period 2017/18 (Invitation period 1^st^ August-31^st^ October) | | | | | | | | | |
| --- | --- | --- | --- | --- | --- | --- | --- | --- | --- |
| Characteristic | Population (n) | Uptake  (%) | OR | 95% CI | p | Adjusted uptake (%) | aOR | 95% CI | p |
| **Overall** | 70,009 | 56.0 | - | - | **-** | 56.1 | - | - | **-** |
| **Sex**  Male  Female | 33,808  36,201 | 54.5  57.4 | Reference  0.89 | 0.86-0.92 | **<0.001** | 55.2  57.1 | Reference  1.08 | 1.05-1.12 | **<0.001** |
| **Age group**  60-64 years  65-69 years  70-74 years | 28,082  19,882  22,045 | 49.1  59.3  61.8 | Reference  1.51  1.67 | 1.45-1.56  1.61-1.73 | **<0.001**  **<0.001** | 49.9  59.3  61.4 | Reference  1.47  1.61 | 1.42-1.53  1.55-1.67 | **<0.001**  **<0.001** |
| **Location**  Urban  Rural | 24,221  43,023 | 55.1  58.1 | Reference  1.13 | 1.10-1.17 | **<0.001** | 55.9  56.8 | Reference  1.04 | 1.01-1.07 | **0.040** |
| **Income deprivation**  Q5 (least deprived)  Q4  Q3  Q2  Q1 (most deprived) | 14,723  14,986  13,926  12,870  10,739 | 63.5  59.5  56.3  52.5  45.8 | Reference  0.85  0.74  0.64  0.49 | 0.81-0.89  0.71-0.79  0.62-0.67  0.46-0.51 | **<0.001**  **<0.001**  **<0.001**  **<0.001** | 63.1  59.3  56.2  52.7  46.6 | Reference  0.85  0.75  0.65  0.50 | 0.81-0.89  0.71-0.78  0.62-0.68  0.47-0.53 | **<0.001**  **<0.001**  **<0.001**  **<0.001** |
| **Ethnic group**  White  Mixed  Asian  Black  Other  Unknown | 59,122  207  483  104  99  9,994 | 58.4  51.7  47.8  47.1  49.5  42.4 | Reference  0.76  0.65  0.64  0.69  0.53 | 0.58-1.00  0.55-0.78  0.43-0.93  0.47-1.04  0.50-0.55 | 0.052  **<0.001**  **0.021**  0.074  **<0.001** | 58.3  53.8  49.9  51.3  52.2  43.5 | Reference  0.84  0.72  0.78  0.77  0.54 | 0.63-1.11  0.60-0.86  0.52-1.16  0.51-1.15  0.52-0.57 | 0.222  **<0.001**  0.214  0.194  **<0.001** |
